# Design, Validation and Annotation of Transcriptome-Wide Oligonucleotide Probes for the Oligochaete Annelid *Eisenia fetida*

**DOI:** 10.1371/journal.pone.0014266

**Published:** 2010-12-08

**Authors:** Ping Gong, Mehdi Pirooznia, Xin Guan, Edward J. Perkins

**Affiliations:** 1 Environmental Services, SpecPro Inc., Vicksburg, Mississippi, United States of America; 2 Department of Psychiatry, School of Medicine, Johns Hopkins University, Baltimore, Maryland, United States of America; 3 Environmental Laboratory, U.S. Army Engineer Research and Development Center, Vicksburg, Mississippi, United States of America; University of North Carolina at Charlotte, United States of America

## Abstract

High density oligonucleotide probe arrays have increasingly become an important tool in genomics studies. In organisms with incomplete genome sequence, one strategy for oligo probe design is to reduce the number of unique probes that target every non-redundant transcript through bioinformatic analysis and experimental testing. Here we adopted this strategy in making oligo probes for the earthworm *Eisenia fetida*, a species for which we have sequenced transcriptome-scale expressed sequence tags (ESTs). Our objectives were to identify unique transcripts as targets, to select an optimal and non-redundant oligo probe for each of these target ESTs, and to annotate the selected target sequences. We developed a streamlined and easy-to-follow approach to the design, validation and annotation of species-specific array probes. Four 244K-formatted oligo arrays were designed using eArray and were hybridized to a pooled *E. fetida* cRNA sample. We identified 63,541 probes with unsaturated signal intensities consistently above the background level. Target transcripts of these probes were annotated using several sequence alignment algorithms. Significant hits were obtained for 37,439 (59%) probed targets. We validated and made publicly available 63.5K oligo probes so the earthworm research community can use them to pursue ecological, toxicological, and other functional genomics questions. Our approach is efficient, cost-effective and robust because it (1) does not require a major genomics core facility; (2) allows new probes to be easily added and old probes modified or eliminated when new sequence information becomes available, (3) is not bioinformatics-intensive upfront but does provide opportunities for more in-depth annotation of biological functions for target genes; and (4) if desired, EST orthologs to the UniGene clusters of a reference genome can be identified and selected in order to improve the target gene specificity of designed probes. This approach is particularly applicable to organisms with a wealth of EST sequences but unfinished genome.

## Introduction

DNA microarrays are now widely used as a powerful tool for studying gene expression and regulation on a global scale and at high throughput [1] as well as for discovery of novel biomarker genes [2], [3]. Currently, there exist three major DNA microarray platforms: spotted cDNA array [4], spotted oligo array (e.g., Agilent microarray [5]), and *in situ* synthesized oligo array (e.g., Affymetrix gene chip [6] and Nimblegen array [7]). Increasing demands on higher throughput, flexibility (customizable), reproducibility, and specificity of microarrays have played on the favorable side for the two oligo array platforms [8], [9]. Technological advances have made it feasible to spot or synthesize *in situ* tens or hundreds of thousands of probes on a glass microscopic slide [9]. To make the best use of the high throughput, hybridization-based oligo array technologies, a comprehensive set of probes has to be designed to interrogate the transcriptome or genes of interest expressed in a particular cell, tissue or the whole organism [10] at a particular moment and circumstance.

Meanwhile, recent development of ultra-high throughput DNA sequencing technologies such as Roche/454, Solexa/Illumina and ABI/SOLiD [11] has enabled *de novo* assembly of transcriptomes or genomes from millions of short sequence reads at a fraction of costs and time required by traditional technologies such as the Sanger capillary-array electrophoresis technology [12]–[14]. The industrialization of both microarray and ultra-high throughput sequencing technologies provides new opportunities to functional genomics research in environmentally relevant organisms like fish and earthworms at a modest cost without the need to run a core genomics facility [9], [15].

Segmented earthworms including *Eisenia spp*. and *Lumbricus spp*. (Fig. S1) play significant ecological roles in maintaining soil fertility and the base of many food chains [16]. They have been used as bioindicators of soil quality and health. *Eisenia fetida*, for instance, is an oligochaete annelid extensively used as test organism in ecotoxicological assessment of toxicant impact on soil ecosystems because of the ease to breed, culture and handle [17]. Unlike other model organisms such as unsegmented nematode *Caenorhabditis elegans* and the polychaete bristle worm *Capitella capitata* (Fig. S1), none of the oligochaete genomes have yet been sequenced despite that many laboratories have isolated genes of interest in the lumbricids and investigated effects of environmental stresses on gene expression using technologies such as suppression subtractive hybridization (SSH), cDNA cloning, Sanger sequencing, and quantitative reverse transcription PCR (qRT-PCR) [18]–[25].

Owen and colleagues (2008) fabricated an 8K cDNA microarray to study biochemical pathways and mechanisms of action associated with developmental and xenobiotic responses in *Lumbricus rubellus* [26], [27]. Previously, we also created a 4K-cDNA microarray to profile gene expression in *E. fetida* as differentially affected by exposure to explosive compounds [28], [29]. We have since expanded our sequencing effort in consideration of the limited transcriptomic coverage of the cDNA probes deposited on the earthworm cDNA array [29]. Recently, we obtained millions of bases of raw sequence reads from two 454 sequencing runs of a normalized full-length double-stranded (ds) cDNA collection prepared from *E. fetida* nerve tissues (an unpublished neurotoxicity study by P. Gong, et al.). The rich EST sequence information of *E. fetida* has motivated us to develop a large set of transcriptome-wide oligo probes that can be used to assemble high-density arrays for transcriptomic profiling. We aimed in this study to identify unique transcripts as targets from the massive amount of *E. fetida* EST reads, to select an optimal and non-redundant oligo probe for each of these targets, and to annotate all of the selected target sequences. To achieve this goal, we developed a streamlined and easy-to-follow approach to the design, validation and annotation of species-specific array probes, particularly for organisms whose genomes have yet to be fully sequenced and annotated.

## Results

**Figure 1:**
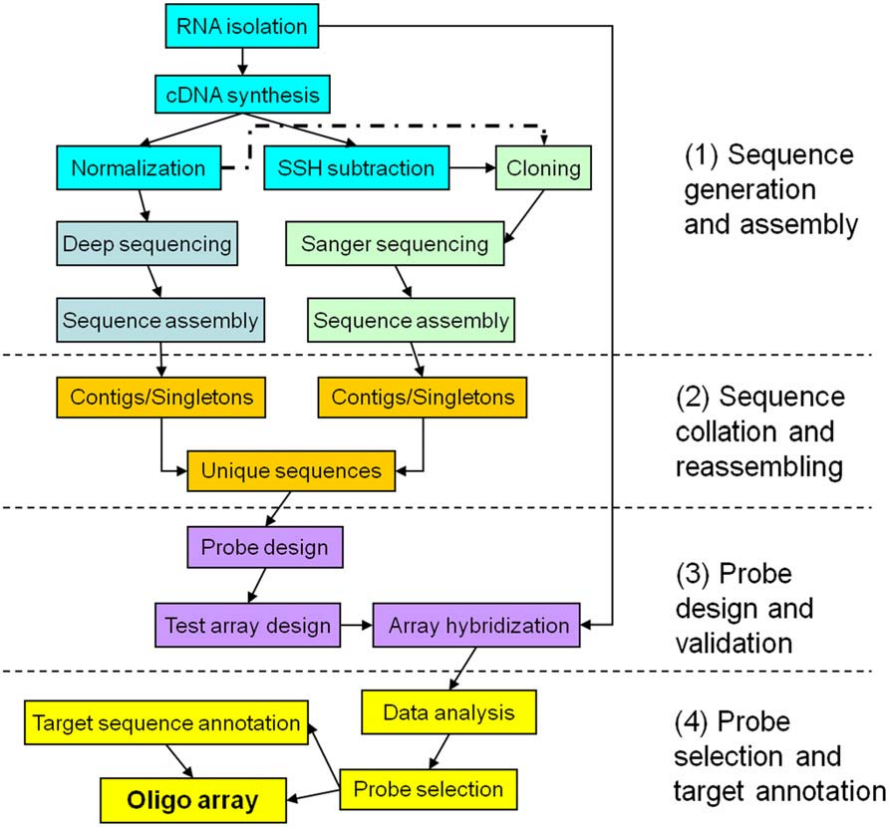
Oligo array design process. Our proposed easy-to-follow approach to design, validation and annotation of microarray oligo probes particularly for organisms with unsequenced genome but having a transcriptomic scale of EST sequences generated with deep sequencing and Sanger sequencing technologies. The SSH subtraction is optional as many researchers often construct normalized cDNA libraries for cloning and Sanger sequencing.

In general, our approach consists of the following four phases (see Figure 1): (1) sequence generation and assembling; (2) sequence collation and reassembling; (3) probe design and testing; and (4) probe selection and target annotation. The whole process begins with generation and assembling of raw EST sequences. A considerable amount of ESTs are required to take advantage of the high throughput nature of microarrays. In phase 2, sequences from all sources are collated and reassembled into contiguous and noncontiguous sequences thereby generating unique target sequences. In phase 3, probes are designed for the unique target sequences, put together on test arrays, and RNA samples are hybridized to the arrays to test the probes. In phase 4, positive probes are identified on the basis of signal intensity of each probe on the test array, and the target sequences of all positive probes are annotated through bioinformatic data mining.

In the present study, we used Sanger sequence reads obtained from our previously published study [18] and high-throughput 454 sequences from our unpublished neurotoxicity study (see **Materials and Methods** for details). We also included 104 *E. fetida* Sanger sequences previously deposited in the NCBI's GenBank by other researchers. Differing from the general guidance illustrated in Figure 1, we used unassembled Sanger reads (see below for explanation) and did not attempt to re-assemble the sequences from different sources (see **Discussion** for explanation). The following describes the remaining steps involved in the process of developing species-specific and transcriptome-wide oligo arrays for *E. fetida* (Figure 1).

### 454 sequence assembly

Two 454 sequencing runs generated 562,327 quality filtered non-directional sequence reads with an average length of 104 bases. These reads were assembled using two proprietary programs, Newbler and SeqMan Pro. Newbler assembled 31,114 contigs (including 682 large contigs ≥500 bases in length) and 157,071 unassembled singletons (Table 1; see Table S1 for Newbler-assembled contigs). SeqMan generated 63,602 contigs (1,996 large contigs ≥500 bases in length) leaving 129,486 unassembled singletons (Table 1; see Table S2 for SeqMan-assembled contigs). Surprisingly, only 448 contigs are identical between the two assemblies based upon results of a BLASTN alignment [30]. This was likely caused by a combination of algorithmic differences in the two assemblers, low sequencing coverage, and shortness of 454 reads. The result may also reflect the inefficiency of both assemblers in handling this particular sequence dataset. Adjustment of parametric settings cannot remedy the algorithmic limitations unless we increase the depth of sequencing coverage and read length [31]. Other assemblers that are designed to handle short reads such as ABySS and SOAP may work better but won't resolve the coverage issue [32].

**Table 1:** Completeness test of two 454 sequence assemblies using BLASTN a.

Assembly	Unique sequences	Significant hits (*E *≤10^**−**5^)	Identity of ≥25 overlapping bases100% identical >95% identical	
Newbler				
Singleton	157,070	493,800	2,109	8,818
Contig	31,114	32,713	579	2,419
Total	188,184	526,513	2,688	11,237
SeqMan				
Singleton	129,486	44,111	917	2,414
Contig	63,602	44,979	339	1,489
Total	193,088	89,090	1,256	3,903

a Each unique 454 singleton or contig was aligned against all other unique sequences within the same assembly.

The low depth of coverage and short length of 454 sequence reads have also created new challenges for probe design, i.e., too many unique sequences (both contigs and singletons). There exists no measure that can compare the accuracy of these two independent assemblies because there is no reference genome to map them to. Hence, we designed two BLASTN-based tests ([30], see **Materials and Methods** for details): a completeness test that examines if an assembler has exhausted all the possibility of assembling contigs under given conditions, and a correctness test that aligns assembled contigs and singletons against longer Sanger sequences. The completeness test suggests that the SeqMan assembly is more thorough than the Newbler assembly because the former has far fewer significant hits (where the expectation value (*E*) ≤10^−5^) and unique sequences that overlap 25 or more bases with other sequences of the same assembly (Table 1). Therefore, the algorithm implemented in Newbler was much more conservative and produced more unique sequences than that in SeqMan [33]. It might not sacrifice assembly accuracy if some of the unique sequences in the Newbler assembly were joined into contigs by lowering the assembly stringency.

On the other hand, some studies suggest that Newbler (the 454 assembler) may outperform SeqMan in terms of precision on short reads produced by 454 sequencing because the SeqMan algorithm has been developed and optimized for relatively longer reads from Sanger sequencing [34]. However, the correctness test indicates that the two assemblies are comparable with regard to significant hits and full-length identity (Table 2). Even though Newbler contigs share a higher degree of identity with unassembled *E. fetida* Sanger sequences, the number of SeqMan contigs of 100 bases or longer that share ≥80% full length identity with the raw *E. andrei* and *Lumbricus spp.* Sanger sequences is twice as many as that of Newbler contigs (Table 2). Results of the two tests indicate that the overall quality of the SeqMan assembly is better than the Newbler assembly.

**Table 2:** Correctness test of two assemblies using BLASTN a.

Assembly	Unique sequences	Significant hits (*E* ≤10^**−**5^)EF EA+LS		Full length identity b≥90% (EF) ≥80% (EA+LS)	
Newbler					
Singleton	157,070	3,207	5,306	400	344
Contig	31,114	1,713	3,613	534	548
Total	188,184	4,920	8,919	934	892
SeqMan					
Singleton	129,486	1,766	3,005	129	207
Contig	63,602	2,813	5,677	593	1,250
Total	193,088	4,579	8,682	722	1,457

a Each unique 454 singleton or contig was aligned against all available Sanger sequences of *Eisenia fetida* (EF) (2231 SSH +104 GenBank dbEST), *Eisenia andrei* (EA) and *Lumbricus spp.* (LS) (1108 EA +17225 LS). If one unique 454 sequence hit more than one Sanger sequences, only the most significant one was counted.

b The full length (≥100 bases) is that of the subject or the query, whichever is shorter.

### Test probe and array design

In test probe design, we assumed both sense and antisense orientations for each target sequence, and four 244K-format test arrays (243,504 spots or features per array) were designed using Agilent's eArray (https://earray.chem.agilent.com/earray/), a web-based program, to probe all available *E. fetida* target ESTs (Table 3). Multiple 60-mer probes were designed to target longer transcripts (≥150 bps) using the best distribution method, whereas a single 60- or 40-mer probe was designed for shorter ones (40∼150 bps) using the best probe method. The difference between test arrays TA-1 and TA-3 or between TA-2 and TA-4 lies that the latter included 40-mer probes. Each array contained 2,105 control spots.

**Table 3:** Design of four 244K-oligo probe test arrays using Agilent's eArray.

						Non-redundant probe within group a			
Target sequence source	Target sequence #	Target length	Probe length	Probe(s)/target	Design method	Sense	antisense	sense	antisense
GenBank dbEST	104	>300	60	4	Best distribution	281	280	281	280
SSH libraries	3144	vary	60	2	Best distribution	5412	5489	5412	5489
454 SeqMan-Singleton	129486	40∼278	60	1	Best probe	(96430)	(96339)	(96430)	(96339)
454 SeqMan-Contig1	40222	<150	60	1	Best probe	(33309)	(33293)	(33309)	(33293)
454 SeqMan-Contig2	18129	150∼300	60	2	Best distribution	31392	31305	31392	31305
454 SeqMan-Contig3	5251	>300	60	4	Best distribution	19502	19509	19502	19509
All above	196336	vary	60	1	Best probe	155244	155213	155244	155213
454 Newbler-Contig	31114	vary	60	1	Best probe	30684	30682	30684	30682
Short unique	26302	40∼59	40	1	Best probe	(23941)	(23907)	23941	23907
Total number of redundant probes among groups						27245	28722	27245	28722
Total number of non-redundant probes in the final test array design						215270	213756	239211	237663
Total number of redundant probes included in the final test array design						26129	27643	2188	3736
Total number of unique and redundant probes in the final test array design						241399	241399	241399	241399
Test Array ID						TA-1	TA-2	TA-3	TA-4
Configuration number of Agilent custom gene expression array in 1x244K-format (catalog no. G4502A)						20022	20023	20024	20025

a A probe group is defined as the collection of probes designed for a specific source of target sequences (e.g., SSH libraries, 454 Newbler-Contig, etc.). Redundant probes were removed within each probe group. Numbers in brackets are the probes excluded from array design because they are either already included in the group called “All above”, or are short 40-mer probes that are excluded from TA-1 and TA-2.

We did not reassemble Sanger and 454 sequences because of the lack of an independent and reliable measure to assess assembly quality and accuracy. Instead, test probes and arrays were designed using eArray to target the entire SeqMan assembly, contigs of the Newbler assembly, and the unassembled Sanger ESTs. We selected the SeqMan assembly due to its higher overall quality relative to the Newbler assembly. The Newbler contigs were selected due to their relatively higher accuracy than the SeqMan contigs as measured by >90% identity to *E. fetida* Sanger sequences (534/31,114 vs. 593/63,602, see Table 3). Unassembled Sanger EST sequences were used instead of assembled ones because (1) the number was so small that it would have little impact on the total probe size; and (2) redundant probes were removed by eArray in case the same probe was designed to target multiple Sanger sequences with significant identity.

### Array hybridization results and probe selection

One pooled *E. fetida* RNA sample representing multiple developmental stages (cocoon, juvenile and adult) along with ten spike-in RNAs of known concentrations was hybridized to each of eight custom-designed 244K oligo arrays (two arrays per design) fabricated by Agilent. Each array was scanned at two PMT gain levels (400 and 500). Data of all eight arrays were deposited in GEO as SuperSeries record GSE16551. The spike-in RNAs were used to construct linear correlation curves, from which signal intensity baseline and saturation levels were established. Any spot with signal intensity no greater than this baseline level was considered insignificantly different from the background. Meanwhile, saturated spots were flagged out because the measurement was unreliable. According to our experimental design, all the 60-mer probes (except for those redundant ones) were tested on four arrays, resulting in eight expression measurements as each array was scanned at two PMT gain settings.

**Figure 2:**
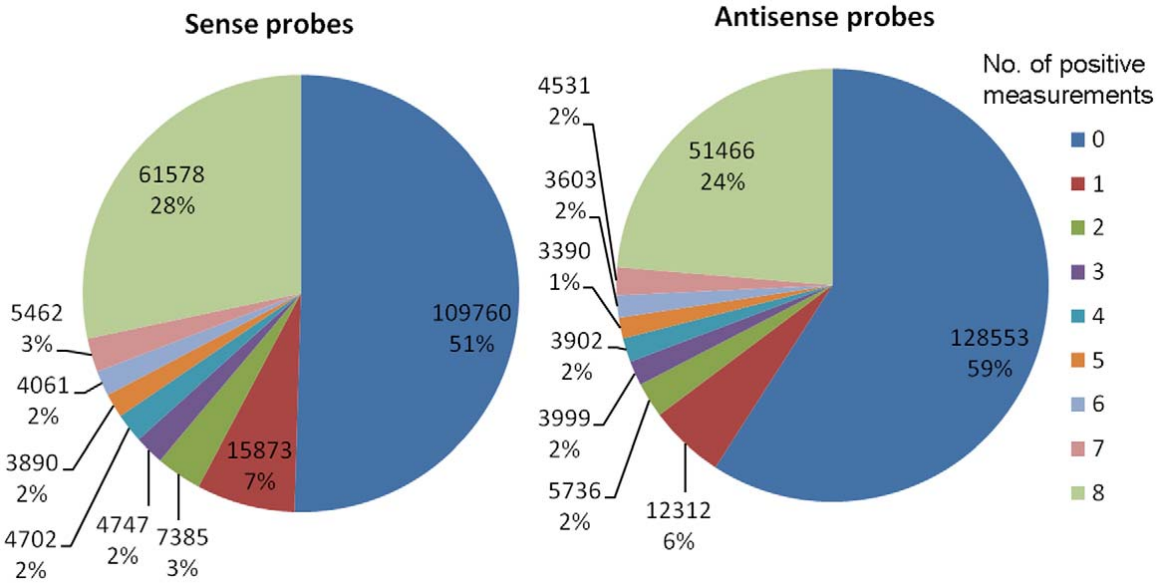
Hybridization results of 60-mer probes. Hybridization results of eArray-designed sense and antisense 60-mer probes showing the number and percentage of probes with 0 to 8 positive measurements. The total number of sense and antisense probes was 217,458 (215,270 unique +2,188 redundant) and 217,492 (213,756+3,736), respectively. Each probe was measured 8 times, i.e., on four arrays (2 array/design) and two PMT gain settings (400 and 500). Results of additional redundant probes included in test array designs TA-1 and TA-2 are not shown because they were measured 4 times if disregarding their repeats.

As shown in Figure 2, over 50% of the designed sense and antisense probes did not produce a single positive signal when hybridized to the pooled cRNA sample, which is in line with our expectation, given one possible orientation for each target EST. There was a slight difference between the scanning results under two different PMT settings, i.e., the lower PMT gain produced more spots with signal intensity close to the background level while the higher PMT gain produced more saturated spots (data not shown). In the most ideal situation where hybridization is determined solely by sequence complementarity, a designed probe would either consistently hybridize if the assumed target orientation is correct or not hybridize at all if wrong. This occurred to the majority (80%) of the designed probes that had either 0 or 8 positive measurements, no matter what target orientation was assumed (Figure 2). However, we saw positive response on some of the arrays for the remaining 20% of the designed probes, possibly due to cross-hybridization of mismatches, non-optimal thermodynamic conditions, or low expression levels, making it necessary to set an arbitrary threshold to determine whether such a probe is a true or false positive one. In this study, we called a positive probe if 75% or more of its measurements were positive.

As one of the objectives of this study was to select a unique probe for a unique target sequence, we define “uniqueness” as (1) one non-redundant EST (unique target sequence) should be probed by one single non-redundant probe (unique probe) and (2) one unique probe should hybridize to one single unique target sequence. As we allowed redundancy with regard to both target sequence and array probe during test array design, the following process was carried out to select for positive and unique probes/targets:

1. Remove probes that are flagged in more than 25% of the measurements (i.e., ≥2/4, 3/8, or 5/16)
2. Remove redundant probes for each assumed target orientation;
3. Remove redundant target sequences and their corresponding probes for each assumed target orientation;
4. Remove redundant probes/targets across orientation as one positive probe of one orientation may be identical to another positive probe of the other orientation;
5. Remove probes that are highly similar to reduce cross-hybridization by setting the threshold at 95% or higher identity of 50 or more consecutive bases using BLASTN;
6. Remove the probe with a lower signal intensity when redundant probes/targets or highly similar probes occur.

For the 60-mer probes, 33,740 (54.5%) sense probes and 28,133 (45.5%) antisense probes passed the process. We also identified 1,668 40-mer probes that responded positively to the hybridized cRNA. Altogether, a total of 63,541 unique probes were validated as positive unique probes. These probe sequences, as well as target EST sequences, can be found in Table S3. These validated probes target transcripts with a total length of 10.4 Mb, covering roughly 1.5% of the estimated 700 Mb *E. fetida* genome ([35] and see www.genomesize.com). In order to estimate how many protein-coding genes these targeted transcripts/transcriptome correspond to, we downloaded from http://genome.jgi-psf.org/Capca1/Capca1.download.ftp.html all the protein-coding gene models (allModels.aa.fasta.gz) of *Capitella capitata*, the closest annelid species with a completed genome sequence (Fig. S1). We queried this database using BLASTX (default settings) and identified 11,580 unique *C. capitata* genes sharing some degree of similarity with 28,150 *E. fetida* transcripts (*E* value ≤10; 3,808 *C. capitata* genes at *E*≤10^−5^). This result suggests that these validated probes may cover more than 1/3 of the 32,415 predicted *C. capitata* gene models. A better estimation of the transcriptomic coverage can be made once the *L. rubellus* genome sequence is released [36]. The remaining 35K *E. fetida* probes may target non-coding regulatory RNA genes including microRNAs, snoRNAs, siRNAs and piRNAs.

The above process ensured the non-redundancy for both probes and targets. It is possible that one sequence could overlap with others since no further assembling was performed for the target sequences from different sources (Table 3). The possibility is greater for target sequences because we have removed highly similar probes in step 5. Further work is required to identify overlapping targets and further refine their probes.

### Probe target annotation

The following databases were queried using various bioinformatic tools to annotate the 63,541 validated and uniquely probed target ESTs: GenBank (BLASTN and BLASTX [30]) and InterPro (InterProScan [37], [38] and PIPA or PIpeline for Protein Annotation [39]) (see **Materials and Methods** for details). The open reading frames (ORFs) present on these target ESTs were translated into 1,074,222 peptides ≥5 amino acids (aa) in length. InterProScan was conducted in the order of peptide length, i.e., from longer to shorter peptide. After having completed a search for peptides longer than 40 aa, we switched to PIPA, a high throughput protein annotation pipeline implemented on high performance computing, due to the low throughput of InterProScan.

We obtained 5,502 BLASTN hits and 9,990 BLASTX hits when we set *E*≤10^−3^ (see Table S4 for search results). At an increased stringency of *E*≤10^−5^, we had 3,771 significant BLASTN hits and 8,234 significant BLASTX hits. InterProScan returned 61,368 hits matching 15,057 target ESTs, whereas PIPA yielded 46,120 hits matching 27,317 target ESTs. Some of the target ESTs had multiple strings of annotation from different algorithms and databases. Overall, 37,439 unique target ESTs have annotation information derived from the above bioinformatic data mining excises.

## Discussion

As outlined in Charles Darwin's last book *The Formation of Vegetable Mould through the Action of Worms* (1881), earthworms are extremely important in soil formation, turnover, aeration, drainage, organic matter breakdown and incorporation, and nutrient availability. These activities have effectively established their roles in maintaining soil fertility and crop production [16]. Meanwhile, earthworms have emerged as one of the best indicators available of soil quality and soil contamination [16], [17]. In many standardized toxicity testing protocols *E. fetida* has been recommended as the test organism [17]. We believe that development of high throughput genomic tools such as species-specific oligo arrays will help advance research in earthworm biology, ecology and ecotoxicology.

We presented here a streamlined and easy-to-follow approach to oligo array design, validation and annotation for the earthworm *E. fetida*. Although this study is unique in the fact that a mixture of EST sequences generated by both next generation and traditional sequencing technologies were used, our approach can be applied to a diverse range of non-model species, for which a large set of EST sequences with transcriptome-wide coverage is available. As the ultra high throughput sequencing technologies become more affordable and acceptable, more and more organisms will be sequenced at the transcriptomic level, leading to an increasing demand for making microarray tools in the foreseeable future.

It is worth noting that different researchers have employed different approaches that best suit their own research goal, as shown in Table 4. For instance, Pariset *et al*. [15] developed a pipeline starting from unannotated, redundant EST sequences to yield oligo probes suitable for *in situ* generation on a DNA chip. Li and colleagues reported a bioinformatics-intensive protocol to generate an optimized set of oligo probes from a minimally redundant but maximally representative list of sequences that were assembled from 270K raw EST data for the rainbow trout (*Oncorhynchus mykiss*) [9]. Magness *et al*. and Wallace *et al*. developed two rhesus macaque-specific oligo arrays with different gene coverage using the same approach but different amounts (20K and 486K, respectively) of ESTs [40], [41]. A rhesus exon sequence was picked as target only if it matched the exon regions of the 23,000 human genes (Human UniGene build 167). Probes were then designed to the region closest to the 3′ untranslated region (UTR) of each rhesus/human ortholog target gene. This approach was feasible largely owing to the high similarity (>99%) between the rhesus and the human genomes, thus limiting its applicability to a wider range of organisms. Although all the designed arrays in Table 4 were tested using mRNA samples isolated from corresponding organisms, none except this study used hybridization results to further eliminate inefficient probes or targets of low copy numbers.

**Table 4:** Comparison of several representative approaches to transcriptome-scale oligo array probe design, validation and annotation based on species-specific EST sequence information.

Organism (common name)	EST # & sequencing technology	Assembler & unique target #	Probe design program	Probe # & length (mer)	Validation	Target EST annotation	Ref.
*Eisenia fetida* (earthworm)	566K, 454 & Sanger	Newbler/SeqMan, N/A a	eArray	See Table 3	Array	BLASNTNBLASTXInterProScanPIPA	This study
*Solea senegalensis* (flatfish)	10K, Sanger	Phrap/Consed, 5K	Tethys	5K, 50–60	Array	BLAST2GO	[52]
*Sparus aurata* (sea bream)	59K, Sanger	Cap3, 20K	eArray	39K, 60	Array & qRT-PCR	BLASTNBLASTX	[50]
*Homo sapiens* (human)	>30K, Sanger	Redundant, 4K	Affymetrix	4K, 25	Array	BLAST	[8]
*Oryza sativa* (rice)	67K, Sanger	Whole genome, varied b	Picky	43K, 50–70	Array	TIGR V5 gene model	[53], [54]
*Macaca mulatta* (monkey)	486K, Sanger	Shotgun genome, 22K	eArray	22K, 60	Array	BLASTNCLUSTALW	[41]
*Myzus persicae* (green peach aphid)	27K, Sanger	Cap3, 10K	eArray	15K, 60	Array	BLASTN BLASTX	[51]
*Oncorhynchus mykiss* (rainbow trout)	227K, Sanger	EST-Ferret, 57K	Array Designer	25K, 65	Array	Gene Index, BLASTX	[9]
*Ovis aries* (sheep)	210K, Sanger	15K Unigene	GoArrays	22K, 40	Array	Annot8r	[15]

a N/A: not available.

b Varied depending on what assembler or estimator was used.

In general, two main goals are to be achieved in building high density arrays for organisms with incompletely sequenced genomes: (1) minimization of the unique target gene number, and (2) oligo probe optimization.

### Minimizing target genes

The first goal requires both sequence assembly and identification of unique gene models so that one probe can be designed to target one unique gene (not an individual EST). Raw EST reads are often assembled into unique contigs or singletons using software such as Cap3, Newbler, Phrap and SeqMan (Table 4). The real challenge is how we can identify a unique gene structure/model from several non-overlapping assembled ESTs if they actually code for different regions of the same gene. There is no perfect solution to this problem but certain degrees of success have been achieved when assembled ESTs are mapped to finished genomes of closely related species [41], [42].

In the current study, we faced an even harder challenge because of the combination of (1) the mixed sources of Sanger and 454 sequences, (2) a few 454 sequencing runs resulting in a low fold-coverage, and (3) the relative short length of 454 reads. It has been recognized that the quality of genome- or transcriptome-scale assembly from shot-gun sequence reads depends heavily on a number of factors such as the depth of coverage and the length and base quality (accuracy) of raw sequence reads [12], [34], [43]. In addition, the cDNA sequences reverse transcribed from RNAs are not genomic DNA sequences, and may be subject to transcriptional and/or post-transcriptional modification (e.g., alternative splicing) [10]. The cDNA collection used for 454 sequencing was generated from dissected earthworm nerve cords (see **Materials and Methods** for details), which inevitably contained peripheral tissues other than neuronal cells. The cDNAs used for Sanger sequencing were isolated from whole worm body tissues. Tissue-specific alternative splicing may have resulted in different RNA transcripts from the same DNA gene [44], and is a plausible reason for the high abundance of unique sequences in the transcriptomic assembly. Lastly, the two 454 sequencing runs were obviously unable to provide a sufficient fold-coverage of either the transcriptome or the genome of *E. fetida*.

In recognition of the aforesaid challenges and limitations, we did not attempt to assemble a complete transcriptome. Instead, we developed two BLASTN-based alignment tests in order to obtain some numerical measurements that would guide us in selecting one of the two 454 assemblies for probe design. In the correctness test, it is obvious that the more unique and long Sanger sequences there are, the more accurate the assessment would be. Due to the limited amount of available *E. fetida* Sanger sequences, we also aligned the 454 assemblies to those of three closely related species, *E. andrei*, *L. rubellus* and *L. terrestris* in order to more effectively evaluate assembly accuracy (Figure 1 and Table 2). We lowered the stringency (identity degree) from 90% to 80% in consideration of genomic divergence. Based on the test results, we have chosen the entire SeqMan assembly and the Newbler contigs for probe design. We will be able to better assess assembly accuracy and design non-redundant probes for every unique gene when the *Lumbricus* genome is released [36], [41], [42].

### Optimizing oligo probes

The second goal of oligo probe optimization is also hard to achieve because little consensus currently exists on how to validate and optimize array probes. Despite some exploratory work [45], [46], the selection of highly specific oligo probes for all targeted genes of interest, while maintaining thermodynamic uniformity at the hybridization temperature, remains a difficult task. There is no perfect solution but to test and select the optimal hybridization conditions that produce the highest signal intensity [41], [45]. Although most researchers adopt oligo probe design programs based on sequence complementarity and *in silico* thermodynamic simulation of hybridization, very few have actually attempted to identify the optimal hybridization conditions [45] and/or the optimal probe from multiple ones designed for the same target gene [40], [41]. Given the current state of the art, we believe that the best strategy for optimizing oligo probes is to empirically test multiple probes and hybridization conditions (particularly temperatures). We only tested multiple probes in the current study.

### Robustness of oligo probe design from ESTs

Different from most of the other studies listed in Table 4, we spent little bioinformatic resources up front. Instead, we allowed all unique sequences to enter the probe design process and designed at least one probe for each one of them on each orientation. We took advantage of the Agilent 244K high density custom array to accommodate the large number of test probes. We also believed that the optimal probes should emerge and prevail from array hybridization results. Intensive annotation efforts were made only on the final 63.5K selected unique targets. This pragmatic approach may miss out quite a few functional genes, which is caused by the technical limitation of array technologies. However, this can be improved as the dynamic range of array detection limits gets widened. The robustness of our approach also lies in that (1) it allows new probes to be easily added and old probes modified or eliminated when new sequence information becomes available, (2) it is not bioinformatics-intensive upfront but does provide opportunities for more in-depth annotation of biological functions for target genes; and (3) if desired, EST orthologs to the UniGene clusters of a reference genome can be identified and selected in phase two (Figure 2) in order to improve the target gene specificity of designed probes [40], [41].

In summary, we have developed a novel approach to the design, validation and annotation of transcriptome-wide oligo probes for an ecotoxicological model organism *E. fetida* from both Sanger and 454 sequences. This approach was particularly tailored for organisms with a wealth of EST sequences but incomplete genome sequence. Our approach is advantageous over others owing to its simplicity (easy-to-follow), low bioinformatics capital, and high robustness. Using this approach one can identify unique transcripts as target sequences, design multiple oligo probes per target, test and select the optimal and unique oligo probes, and annotate the final selected probed target sequences.

This work constitutes part of a larger effort of our earthworm toxicogenomics program. Based on the results from this work, we have constructed two Agilent 60-mer oligo custom arrays, a 15K-feature array (AMADID# 021219) and a 44K-feature array (AMADID# 022725). Both arrays have been used as powerful functional genomics tools for profiling gene expression response to toxicity stressors and for discovery of novel biomarker genes [47]. It is also our intention to share the validated array probes with other researchers in the earthworm research community. Those who are interested in studying functional genomics of *E. fetida* may contact the corresponding author for access to the current validated probe set and future updates.

Our future work will focus on generating more and longer ESTs (average length  = 400∼500 bases) using the upgraded 454 GS FLX system coupled with the Titanium chemistry in order to increase the depth of transcriptomic coverage, and on mapping *E. fetida* ESTs to the already completed bristle worm (*Capitella capitata*) genome and the *Lumbricus rubellus* genome (an ongoing sequencing effort [36]; see http://xyala.cap.ed.ac.uk/Lumbribase/index.shtml for details). These data will be incorporated into an *E. fetida* genomic database. Our ultimate goal is to identify and refine expressed genes (UniGene) while improve the specificity and sensitivity of oligo probes designed to target each and every one of them.

## Materials and Methods

### RNA isolation

A continuous *E. fetida* culture was maintained in our laboratory as previously described [18]. Mature worms with a visible clitelum (0.4∼0.6 g/worm) were used for preparing cDNA samples for sequencing and cRNA probes for array hybridization. Juveniles and cocoons were also used for hybridization. Both mature worms and juveniles were depurated on moistened filter paper overnight before being rinsed in RNase-free water and snap-frozen in liquid nitrogen. Cocoons were washed using RNase-free water and snap-frozen. Frozen worms/juveniles/cocoons were fixed in RNAlater®-ICE (Ambion) at −80°C for 24 hr and then stored at −20°C. Total RNA was extracted from either the fixed whole worm/juvenile/cocoon (for SSH cDNA library construction and array hybridization) or the nerve cord tissue dissected from the fixed mature worms (for 454 sequencing) using RNeasy kits (Qiagen).

### Preparation of *E. fetida* cDNA samples for sequencing

Two cDNA libraries for Sanger sequencing were constructed from mature worms in our previous study [18] using an approach that involved suppressive subtraction, normalization and PCR amplification (SSH, Clontech), insertion of *Taq* polymerase-amplified PCR products into a plasmid vector (TOPO® cloning, Invitrogen), PCR-amplification and purification of positive inserts, and sequencing on a 16-capillary ABI PRISM® 3100 Genetic Analyzer.

A full-length ds-cDNA collection for 454 sequencing was prepared in an unpublished neurotoxicity study (P. Gong, et al.) by pooling total RNA samples (475 ng) isolated from the dissected nerve tissue of 44 adult worms, synthesizing full length ds-cDNAs using SMART™ (Switching Mechanism at 5′ End of RNA Template) technology (Clontech) [48], and normalizing the cDNA pool where known adaptor sequences were incorporated at both ends of cDNA using TRIMMER (Evrogen). TRIMMER is a cDNA normalization kit based on degradation of ds-fraction formed during cDNA reassociation by a unique Duplex-Specific Nuclease (DSN) enzyme [49]. The final cDNA sample of 125 ng/µl in 42 µl RNase-free water (A_260_/A_280_ = 1.84) was supplied to Roche 454 Life Sciences (Branford, CT) for sequencing on a Genome Sequencer 20 (GS20) system.

### *E. fetida* Sanger sequences

As previously reported [18], we cloned a total of 4,032 cDNAs from the two SSH libraries, sequenced 3,816 positive clones, and trimmed off contamination of low quality ends and vectors/adaptors using CodonCode Aligner. A total of 3,144 good quality sequences with an average length of 310 bases (deposited in GenBank db EST under accession# EH669363-EH672369 and EL515444-EL515580) were used in the present study (Table 3).

### 454 sequencing and sequence assembly

Two sequencing runs were performed at 454 Life Sciences on the normalized shot-gun cDNA sample using the 454 GS20, which generated 562,327 quality filtered non-directional sequence reads. This sequence dataset was deposited in NCBI's Short Read Archive under submission# SRA009433. Two assemblers, Newbler and SeqMan Pro (build 7.2 with default settings), were used in order to compare their performance in assembling the 454 sequence reads after trimming off adaptor and primer sequences. The Newbler assembly was conducted by 454 Life Sciences.

### Comparison between the Newbler assembly and the SeqMan assembly

Two BLASTN-based [30] tests were conducted to compare the accuracy of the two assemblies: (1) a completeness test, in which an assembled contig or singleton was aligned against all other contigs and singletons within the same assembly to assess if there were any more contigs that could be assembled (Table 1); and (2) a correctness test where each assembly were aligned against the unassembled Sanger ESTs of *E. fetida* and three closely related lumbricids to evaluate the accuracy of assembled 454 contigs (Table 2). In the completeness test, we used identity of 25 or more overlapping bases between any pair of sequences as the sole endpoint to evaluate whether these two sequences could be merged. We also listed the number of alignments with significant *E* values (*E*≤10^−5^) to show how many sequences in the assembly shared high similarity. In the correctness test, the assessment endpoint was the number of assembled 454 sequences sharing ≥90% or ≥80% identity of the full length (≥100 bases) with the Sanger sequences of *E. fetida* or *E. andrei* and *Lumbricus spp.*, respectively. The full length is defined as that of the subject or the query, whichever is shorter. Only the alignment with the highest similarity was counted if one assembled 454 sequence matched more than one Sanger sequences. The number of significant hits (alignments with *E*≤10^−5^) was also listed in Table 2.

### Oligo probe design

Agilent's eArray was employed to design oligo probes and assemble 244K-feature test arrays [41], [50], [51]. Both sense and anti-sense orientations were assumed for each target sequence. Two or four 60-mer probes were designed using the best distribution method to cover the full length of target transcripts for those longer than 150 bps (or those in the SSH libraries) and those longer than 300 bps, respectively. A single 60-mer probe was designed to target shorter sequences (60∼150 bps) following the best probe methodology. We also designed 40-mer probes representing ESTs shorter than 60 bps. There were 2,105 control spots, which included 10 quality control probes targeting 10 different spike-in RNAs with each probe replicating 60 times. There were 241,399 feature spots (*E. fetida* probes) and the number of duplicated features varied from 2K to 27K, depending on how many spots were left after accommodating the non-redundant probes (Table 3).

### Test array hybridization

Custom-designed test arrays in the format of 1x244K were purchased from Agilent. Arrays were manufactured with Agilent's Sure-Print Inkjet technology. A pooled RNA sample isolated from *E. fetida* adults, juveniles and cocoons was used for hybridization. Sample cRNA synthesis, labeling, hybridization and microarray processing were performed according to manufacturer's protocol “One-Color Microarray-Based Gene Expression Analysis” (version 1.0). The Agilent One-Color Spike-Mix (part number 5188–5282) was diluted 5,000-fold and 5 µL of the diluted spike-in mix was added to 500 ng of each of the total RNA samples prior to labeling reactions. The spike-in mix consisted of a mixture of 10 in vitro synthesized, polyadenylated transcripts derived from the Adenovirus E1A gene. The labeling reactions were performed using the Agilent Low RNA Input Linear Amplification Kit in the presence of cyanine 3-CTP. The labeled cRNA from each labeling reaction was hybridized to eight individual arrays (two arrays per design) at 65°C for 17 hours using Agilent's Gene Expression Hybridization Kit. After washing, the arrays were scanned at PMT levels 400 and 500 using GenePix 4200AL scanner.

### Microarray data analysis

GenePix Pro 6 (Molecular Devices, Sunnyvale, CA) was used to process microarray images and to acquire signal intensity data. The background-subtracted median signal intensity (SI) was exported and used for further data analysis. A threshold SI (mean +2× standard deviation of the lowest three spike-in RNAs) was set for each array. Any spot with a SI smaller than the threshold was flagged as absent, whereas others were considered present and thus probes deposited on these spots were regarded as positive probes. Negative control spots were also checked to make sure their SI did not exceed those of the lowest spike-in RNAs. Spots with an SI ≥65,000 were considered “saturated” and were hence flagged out.

### Target sequence annotation

The GenBank non-redundant protein and nucleotide databases were queried using BLASTX and BLASTN algorithms [30] for all the selected, unique and probed target sequences with a cutoff setting of Expectation (*E*) value at 0.001. The InterPro databases were also searched using a stand-alone InterProScan program [37], [38] to identify protein sequences similar to ORFs translated from our target sequences with a cutoff ORF length of 5 amino acids. We wrote a Perl script allowing us to utilize EMBOSS (http://emboss.sourceforge.net/) getorf program to create ORFs for each EST sequence. Meanwhile, PIPA, an automated pipeline implemented on high performance computing [39], was also employed to predict protein functions for the ORFs remaining after InterProScan.

## Supporting Information

Figure S1 Taxonomic tree. Evolutionary distances among four earthworm species (Eisenia fetida, Eisenia andrei, Lumbricus rubellus, and Lumbricus terrestris), the marine polychaete bristle worm Capitella capitata and the nematode worm Caenorhabditis elegans.
(0.08 MB TIF)

Table S1 Newbler assembled contigs. Name and sequence of 31,114 contigs assembled using Newbler, a 454 assembler.
(6.20 MB TXT)

Table S2 SeqMan assembled contigs. Name and sequence of 63,602 contigs assembled using SeqMan Pro (build 7.2 with default settings).
(11.74 MB TXT)

Table S3 Validated oligo probes and their target sequences. Name and sequence of validated 60-mer and 40-mer probes and their target ESTs, as well as their length and hybridization signal intensity (mean and coefficient of variance).
(5.34 MB ZIP)

Table S4 Annotation of validated target ESTs. Annotation information obtained for the 65,365 positive target ESTs (before removing highly similar 60-mer probe sequences), which includes query results from BLASTN, BLASTX, InterProScan and PIPA.
(9.89 MB ZIP)

## References

[1] Gershon D (2005). DNA microarrays: more than gene expression.. Nature.

[2] Chu W, Ghahramani Z, Falciani F, Wild DL (2005). Biomarker discovery in microarray gene expression data with Gaussian processes.. Bioinformatics.

[3] Forrest MS, Lan Q, Hubbard AE, Zhang L, Vermeulen R (2005). Discovery of novel biomarkers by microarray analysis of peripheral blood mononuclear cell gene expression in benzene-exposed workers.. Environ Health Perspect.

[4] Duggan DJ, Bittner M, Chen Y, Meltzer P, Trent JM (1999). Expression profiling using cDNA microarrays.. Nat Genet.

[5] Hughes TR, Mao M, Jones AR, Burchard J, Marton MJ (2001). Expression profiling using microarrays fabricated by an ink-jet oligonucleotide synthesizer.. Nat Biotechnol.

[6] Lipshutz RJ, Fodor SP, Gingeras TR, Lockhart DJ (1999). High density synthetic oligonucleotide arrays.. Nat Genet.

[7] Nuwaysir EF, Huang W, Albert TJ, Singh J, Nuwaysir K (2002). Gene expression analysis using oligonucleotide arrays produced by maskless photolithography.. Genome Res.

[8] Borup RH, Toppo S, Chen YW, Teslovich TM, Lanfranchi G (2002). Development and production of an oligonucleotide MuscleChip: use for validation of ambiguous ESTs.. BMC Bioinformatics.

[9] Li W, Olohan L, Williams D, Hughes M, Gracey A (2009). Application of ESTs in microarray analysis.. Methods Mol Biol.

[10] Ruan Y, Le BP, Ng HH, Liu ET (2004). Interrogating the transcriptome.. Trends Biotechnol.

[11] Ansorge WJ (2009). Next-generation DNA sequencing techniques.. N Biotechnol.

[12] Sundquist A, Ronaghi M, Tang H, Pevzner P, Batzoglou S (2007). Whole-genome sequencing and assembly with high-throughput, short-read technologies.. PLoS ONE.

[13] Hillier LW, Marth GT, Quinlan AR, Dooling D, Fewell G (2008). Whole-genome sequencing and variant discovery in *C. elegans*.. Nat Methods.

[14] Shaffer C (2007). Next-generation sequencing outpaces expectations.. Nat Biotechnol.

[15] Pariset L, Chillemi G, Bongiorni S, Spica VR, Valentini A (2009). Microarrays and high-throughput transcriptomic analysis in species with incomplete availability of genomic sequences.. N Biotechnol.

[16] Edwards CA, Edwards CA (2004). The importance of earthworms as key representatives of the soil fauna.. Earthworm Ecology.

[17] Reinecke AJ, Reinecke SA, Edwards CA (2004). Earthworms as test organisms in ecotoxicological assessment of toxicant impacts on ecosystems.. Earthworm Ecology.

[18] Pirooznia M, Gong P, Guan X, Inouye LS, Yang K (2007). Cloning, analysis and functional annotation of expressed sequence tags from the Earthworm Eisenia fetida.. BMC Bioinformatics.

[19] Demuynck S, Grumiaux F, Mottier V, Schikorski D, Lemiere S (2007). Cd/Zn exposure interactions on metallothionein response in *Eisenia fetida* (Annelida, Oligochaeta).. Comp Biochem Physiol C Toxicol Pharmacol.

[20] Myohara M, Niva CC, Lee JM (2006). Molecular approach to annelid regeneration: cDNA subtraction cloning reveals various novel genes that are upregulated during the large-scale regeneration of the oligochaete, Enchytraeus japonensis.. Dev Dyn.

[21] Brulle F, Mitta G, Cocquerelle C, Vieau D, Lemiere S (2006). Cloning and real-time PCR testing of 14 potential biomarkers in *Eisenia fetida* following cadmium exposure.. Environ Sci Technol.

[22] Spurgeon DJ, Svendsen C, Lister LJ, Hankard PK, Kille P (2005). Earthworm responses to Cd and Cu under fluctuating environmental conditions: a comparison with results from laboratory exposures.. Environ Pollut.

[23] Lee MS, Cho SJ, Tak ES, Lee JA, Cho HJ (2005). Transcriptome analysis in the midgut of the earthworm (*Eisenia andrei*) using expressed sequence tags.. Biochem Biophys Res Commun.

[24] Ricketts HJ, Morgan AJ, Spurgeon DJ, Kille P (2004). Measurement of annetocin gene expression: a new reproductive biomarker in earthworm ecotoxicology.. Ecotoxicol Environ Saf.

[25] Sugimoto M, Nakajima N (2001). Molecular cloning, sequencing, and expression of cDNA encoding serine protease with fibrinolytic activity from earthworm.. Biosci Biotechnol Biochem.

[26] Owen J, Hedley BA, Svendsen C, Wren J, Jonker MJ (2008). Transcriptome profiling of developmental and xenobiotic responses in a keystone soil animal, the oligochaete annelid *Lumbricus rubellus*.. BMC Genomics.

[27] Svendsen C, Owen J, Kille P, Wren J, Jonker MJ (2008). Comparative transcriptomic responses to chronic cadmium, fluoranthene, and atrazine exposure in *Lumbricus rubellus*.. Environ Sci Technol.

[28] Gong P, Guan X, Inouye LS, Pirooznia M, Indest KJ (2007). Toxicogenomic analysis provides new insights into molecular mechanisms of the sublethal toxicity of 2,4,6-trinitrotoluene in *Eisenia fetida*.. Environ Sci Technol.

[29] Gong P, Guan X, Inouye L, Deng Y, Pirooznia M (2008). Transcriptomic analysis of RDX and TNT interactive sublethal effects in the earthworm *Eisenia fetida*.. BMC Genomics.

[30] Johnson M, Zaretskaya I, Raytselis Y, Merezhuk Y, McGinnis S (2008). NCBI BLAST: a better web interface.. Nucleic Acids Res.

[31] Schatz MC, Delcher AL, Salzberg SL (2010). Assembly of large genomes using second-generation sequencing.. Genome Res.

[32] Miller JR, Koren S, Sutton G (2010). Assembly algorithms for next-generation sequencing data.. Genomics.

[33] Vera JC, Wheat CW, Fescemyer HW, Frilander MJ, Crawford DL (2008). Rapid transcriptome characterization for a nonmodel organism using 454 pyrosequencing.. Mol Ecol.

[34] Chaisson MJ, Pevzner PA (2008). Short read fragment assembly of bacterial genomes.. Genome Res.

[35] Gregory TR, Nicol JA, Tamm H, Kullman B, Kullman K (2007). Eukaryotic genome size databases.. Nucleic Acids Res.

[36] Sturzenbaum SR, Andre J, Kille P, Morgan AJ (2009). Earthworm genomes, genes and proteins: the (re)discovery of Darwin's worms.. Proc Biol Sci.

[37] Mulder NJ, Apweiler R, Attwood TK, Bairoch A, Bateman A (2005). InterPro, progress and status in 2005.. Nucleic Acids Res.

[38] Quevillon E, Silventoinen V, Pillai S, Harte N, Mulder N (2005). InterProScan: protein domains identifier.. Nucleic Acids Res.

[39] Yu C, Zavaljevski N, Desai V, Johnson S, Stevens FJ (2008). The development of PIPA: an integrated and automated pipeline for genome-wide protein function annotation.. BMC Bioinformatics.

[40] Magness CL, Fellin PC, Thomas MJ, Korth MJ, Agy MB (2005). Analysis of the *Macaca mulatta* transcriptome and the sequence divergence between Macaca and human.. Genome Biol.

[41] Wallace JC, Korth MJ, Paeper B, Proll SC, Thomas MJ (2007). High-density rhesus macaque oligonucleotide microarray design using early-stage rhesus genome sequence information and human genome annotations.. BMC Genomics.

[42] Gnerre S, Lander ES, Lindblad-Toh K, Jaffe DB (2009). Assisted assembly: how to improve a de novo genome assembly by using related species.. Genome Biol.

[43] Diguistini S, Liao NY, Platt D, Robertson G, Seidel M (2009). De novo genome sequence assembly of a filamentous fungus using Sanger, 454 and Illumina sequence data.. Genome Biol.

[44] Noh SJ, Lee K, Paik H, Hur CG (2006). TISA: tissue-specific alternative splicing in human and mouse genes.. DNA Res.

[45] Chou HH, Trisiriroj A, Park S, Hsing YC, Ronald PC (2009). Direct calibration of PICKY-designed microarrays.. BMC Bioinformatics.

[46] Leparc GG, Tuchler T, Striedner G, Bayer K, Sykacek P (2009). Model-based probe set optimization for high-performance microarrays.. Nucleic Acids Res.

[47] Li Y, Wang N, Perkins EJ, Zhang C, Gong P (2010). Identification and optimization of classifier genes from multi-class earthworm microarray dataset.. PLoS One.

[48] Zhu YY, Machleder EM, Chenchik A, Li R, Siebert PD (2001). Reverse transcriptase template switching: a SMART approach for full-length cDNA library construction.. Biotechniques.

[49] Zhulidov PA, Bogdanova EA, Shcheglov AS, Vagner LL, Khaspekov GL (2004). Simple cDNA normalization using kamchatka crab duplex-specific nuclease.. Nucleic Acids Res.

[50] Ferraresso S, Vitulo N, Mininni AN, Romualdi C, Cardazzo B (2008). Development and validation of a gene expression oligo microarray for the gilthead sea bream (*Sparus aurata*).. BMC Genomics.

[51] Ramsey JS, Wilson AC, de VM, Sun Q, Tamborindeguy C, Winfield A (2007). Genomic resources for Myzus persicae: EST sequencing, SNP identification, and microarray design.. BMC Genomics.

[52] Cerda J, Mercade J, Lozano JJ, Manchado M, Tingaud-Sequeira A (2008). Genomic resources for a commercial flatfish, the Senegalese sole (*Solea senegalensis*): EST sequencing, oligo microarray design, and development of the Soleamold bioinformatic platform.. BMC Genomics.

[53] Chou HH, Hsia AP, Mooney DL, Schnable PS (2004). Picky: oligo microarray design for large genomes.. Bioinformatics.

[54] Jung KH, Dardick C, Bartley LE, Cao P, Phetsom J (2008). Refinement of light-responsive transcript lists using rice oligonucleotide arrays: evaluation of gene-redundancy.. PLoS ONE.

