# Supplementary figures and images for: Design, Validation and Annotation of Transcriptome-Wide Oligonucleotide Probes for the Oligochaete Annelid Eisenia fetida
Source: PLoS One. 2010 Dec 8;5(12):e14266. doi: 10.1371/journal.pone.0014266 (PMC2999564; doi:10.1371/journal.pone.0014266)

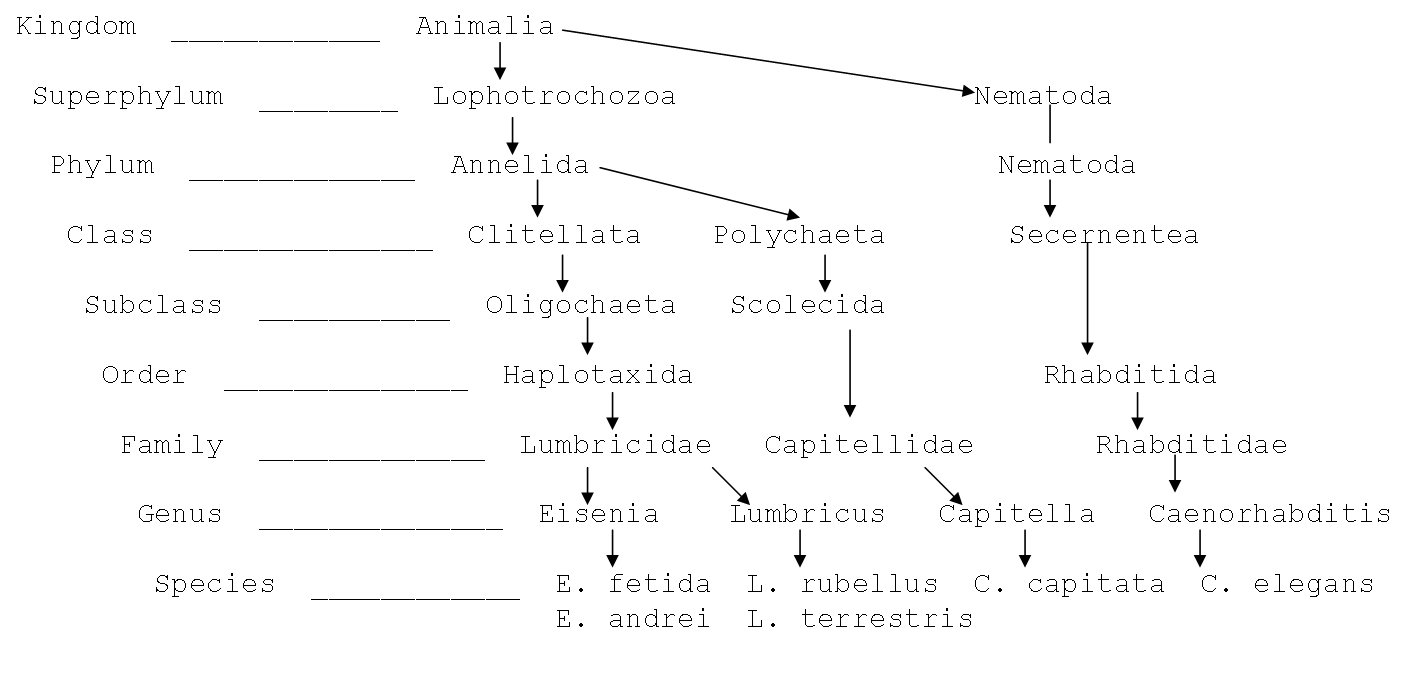

Supplement: Figure S1 — Taxonomic tree. Evolutionary distances among four earthworm species (Eisenia fetida, Eisenia andrei, Lumbricus rubellus, and Lumbricus terrestris), the marine polychaete bristle worm Capitella capitata and the nematode worm Caenorhabditis elegans. (0.08 MB TIF) [file pone.0014266.s001.tif]
